# Communicable Diseases: A Global Perspective

**DOI:** 10.4269/ajtmh.16-0624

**Published:** 2016-10-05

**Authors:** Philip J. Rosenthal

**Affiliations:** 1 Department of Medicine University of California, P.O. Box 0811 San Francisco, CA 94946 E-mail: philip.rosenthal@ucsf.edu

***Communicable Diseases: A Global Perspective***. Roger Webber, 2016. 5th Edition (20th Anniversary Edition). 340 pp. Boston MA: CABI. ISBN 978-1-78064-742-5

This is the 5th edition and 20th anniversary edition of *Communicable Diseases: A Global Perspective*, a single-author book by Roger Webber. The book covers a huge topic, but is compact, 340 paperback pages in the flesh, and also available as an e-book. It is small enough to carry around without risk of injury, and is reasonably priced for a textbook. At first glance one learns that the book has an unusual and clever organization, with diseases organized not by pathogen taxonomy but rather based on means of transmission or other unifying features, for example, chapters entitled “Diseases of Poor Hygiene,” “Diseases of Soil Contact,” and “Insect-borne Diseases.” This organization seems not only a positive, as it offers a more interesting read than does the usual pathogen-based organization, especially for those not focused on pathogen biology, but also a negative, as it is hard to find diseases of interest. Indeed, this should not be seen as a reference book on tropical pathogens; there are much thicker books to fill that niche. Rather, this book offers easily portable one-stop shopping covering global infectious diseases. The book should not be the only text covering tropical infectious diseases on the shelf of a tropical medicine clinician or researcher, but it offers a broad and approachable introduction on this large topic for students, tropical medicine health workers, and others interested in international infectious diseases.

The book opens with 80 pages on principles of communicable diseases and public health. These include discussions of basic principles of communicable diseases, disease control principles and methods, control strategies, and public health. These chapters cover a lot of ground efficiently, will be particularly useful to students, and are a strong feature of the book.

The remainder of the book entails 13 chapters and a total of 113 subsections, most covering a specific infectious disease, and some covering important topics not linked to a single disease. The chapters are organized, as mentioned above, by various criteria that link the diseases, such as how they are transmitted (“Diseases Transmitted by Body Fluids”), shared clinical features (“Respiratory Diseases and Other Airborne-Transmitted Infections”), or special factors (“Pregnancy and Infection”). This organization makes good sense, but since it is different than what we usually see in textbooks, it takes some getting used to. If you want to find meningococcal meningitis, where do you look? It is under “Respiratory Diseases and Other Airborne-Transmitted Infections.” Is leptospirosis a disease of water contact or a zoonosis? In this book it is the latter. Why is ascariasis under “Diseases of Soil Contact”? There is an index to help, but as is often the case with textbooks, the index sends you to many locations, not only the main section focused on the disease of interest. So, the book will work best for those interested in reading through sections on multiple diseases with similar features, but quickly finding a discussion on a specific disease may be a bit frustrating.

Organizational challenges aside, how well does the content of the book handle this big topic? I am happy to say, generally quite well. Many sections are very short, and for dozens of diseases these short sections do a good job of offering a brief summary. The big diseases get a bit more space, for example, 7 pages for tuberculosis, 10 pages for malaria, and 6 pages for human immunodeficiency virus (HIV) infection. A book such as this one cannot help but be criticized for its choice of emphasis. A search for cysticercosis, a common cause of epilepsy in many countries and the most common serious helminth-caused illness seen in American hospitals, led to only two short sentences on this important syndrome. In contrast, plague, a rather rare disease, merited nearly 6 pages.

For some fast-moving topics, the book is a bit behind. For HIV, global statistics are given for 2007, and remarkably, there is very limited mention of antiretroviral therapy, which is now widely and increasingly available for treatment and control, even in many of the poorest countries. The malaria chapter is more up-to-date, with appropriate consideration of modern artemisinin-based combination therapy regimens. “New and Emerging Diseases” warrant a chapter that focuses more on concepts than specific diseases. This seems appropriate, as who can predict which disease will emerge next? A disappointment is that most of the tables and figures seem to have been taken from the last edition of the book. So, Zika does not make it to the table listing 18 important arbovirus infections, and both tables and maps are often well out-of-date.

*Communicable Diseases: A Global Perspective* is reader friendly, with large print, appealing format, and many figures and tables. The book version is in black, white, and shades of blue, but even without full color, the figures generally communicate well. Indeed, many are simple line drawings (including the obligatory life cycles), and these often are more helpful than the very complicated images that can be found on the Internet. A few big tables are particularly valuable. A list of disability-adjusted life years and deaths from leading diseases is a great idea, although the 2004 World Health Organization data are terribly out-of-date for many diseases. A table on diseases transmitted by ticks will be great for testing the most obsessive infectious disease minds (Bhanja, Dhori, Dugbe, Issyk-Kul, and a few dozen more obscure diseases). The last chapter is a massive table listing hundreds of diseases and organisms (if you are not familiar with Jurona virus or Karshi virus or Kasokero virus or many more, they are all here). These big tables are cursory, with just a few words on clinical features and means of transmission for each illness, but they can offer a starting point for additional reading. Also, they serve to teach students and remind others of the vast extent of tropical infectious diseases. As many exotic pathogens have the potential to explode (consider Ebola and chikungunya and Zika in recent years), a good argument can be made for listing diseases that are now obscure, but might soon be in the headlines.

Overall, although serious consideration of the massive topic of global infectious diseases exposes some limitations of this short textbook, when considered as a resource for those new to the field or only seeking brief descriptions of concepts and illnesses, it hits the mark. Those who read it cover to cover and others who peruse specific topics of interest will be rewarded with a valuable introduction to international communicable diseases. And, when they forget whether one gets strongyloidiasis from eating the wrong meal or stepping in the wrong place (it is the latter), *Communicable Diseases: A Global Perspective* will be available to succinctly set the record straight.

